# Effect of Uncomplicated Diabetes Mellitus on Acute Respiratory Distress Syndrome Among COVID-19 Patients in Aseer Region, Saudi Arabia

**DOI:** 10.7759/cureus.31793

**Published:** 2022-11-22

**Authors:** Muneer J Bhat, Yazan A Almaker, Amjd S Algarni, Zyad M Alashqan, Fares Ali M Aljarallah, Ahmad AlIbrahim, Talal K Alshehri, Ziyad S Al-Asmari, Abdulqader Alshahrani, Abdullah Alsalem, Adel H Alfaifi, Ayman M Hammad

**Affiliations:** 1 Department of Surgery, King Khalid University, Abha, SAU; 2 College of Medicine, King Khalid University, Abha, SAU; 3 Intensive Care Unit, Aseer Central Hospital, Abha, SAU

**Keywords:** complications, healthy patients, relations, ards, uncomplicated diabetes, covid-19

## Abstract

Background: Coronavirus disease 2019 (COVID-19) is an infectious disease caused by severe acute respiratory syndrome coronavirus 2 (SARS-CoV-2; an ssRNA virus), which mainly affects the respiratory system but can also cause damage to other body systems. Acute respiratory distress syndrome (ARDS) is a serious complication of COVID-19 that requires early recognition and comprehensive management. ARDS is a diffuse inflammatory process that causes diffuse alveolar damage in the lung.

Aim: The study aimed to assess the effect of uncomplicated diabetes mellitus on ARDS among COVID-19 patients in the Aseer region.

Methodology: A retrospective cohort study was conducted in Aseer Central Hospital between July 10, 2021 to Jan 15, 2022 where confirmed inpatient COVID-19 cases in the Aseer region were classified into two groups. The first group was diabetic patients without any diabetes-related complications and confirmed for COVID-19 infection (diabetes group). The second group was confirmed COVID-19 patients free from any chronic disease. Extracted data included patients’ diabetes status, medical history, socio-demographic data, COVID-19 infection data and vaccination, experienced signs and symptoms, tachypnea, use of accessory muscles of respiration, nasal flaring, grunting, cyanosis, need for hospitalization, need for mechanical ventilation and ICU admission.

Results: The study included 144 patients with uncomplicated diabetes and 323 healthy patients with COVID-19 infection. The mean age of the diabetic group was 65.4 ± 12.9 years old compared to 40.2 ± 11.9 years old for the healthy group. Only one case of the diabetic group was vaccinated against COVID-19 at the study period versus two cases of the healthy group (P=.925). Also, 14 (9.7%) of the diabetic group were contacted with confirmed COVID-19 cases in comparison to 44 (13.6%) healthy cases (P=.238). A total of five (3.5%) diabetic cases needed mechanical ventilation during hospitalization compared to 23 (7.1%) healthy cases with no statistical significance (P=.125). Also, 12 (8.3%) diabetic cases admitted to ICU versus 42 (13%) of healthy cases (P=.145).

Conclusions: In conclusion, there is a great controversy regarding the effect of diabetes on the progression of COVID-19 infection to ARDS. The current study showed that there was no significant difference between diabetic and healthy COVID-19 infected cases regarding ARDS related clinical factors mainly need of ICU admission and mechanical ventilation.

## Introduction

Coronavirus disease 2019 (COVID-19) is caused by the severe acute respiratory syndrome coronavirus 2 (SARS-CoV-2), capable of producing severe symptoms and in some cases death, especially in older people and those with underlying health conditions [1]. COVID-19 is an infectious disease that mainly attacks the respiratory system but also could affect other human systems [2]. Acute respiratory distress syndrome (ARDS) is a reported serious consequence of COVID-19 that needs rapid diagnosis and proper management [3]. ARDS is featured by a diffuse inflammatory process that causes massive alveolar injury in the lung [4]. ARDS is characterized by disturbance of the alveolar coating and capillary endothelium, with alveolar oedema due to protein leakage combined with following fibrosis, and a resultant ventilation-perfusion mismatch [5]. Patients with trauma, pneumonia, sepsis, aspiration, and massive transfusion are at high risk of developing ARDS [6,7].

Diabetes mellitus (DM) is an endocrinological disorder characterized by a state of hyperglycaemia due to insulin resistance, insulin insufficiency, or excessive glucagon secretion [8,9]. Diabetic patients, severe obesity, cardiovascular disease, and hypertension showed increased risk of poor clinical outcome with COVID-19 infection [10-13]. A case cohort including 70,000 persons with confirmed COVID-19 conducted by the Chinese Centre for Disease Control and Prevention found increased mortality in patients with diabetes, increasing from 2.3% in the general population to 7.3% among diabetics [14].

On the other hand, research showed that well-controlled blood glucose was associated with better outcomes among patients with COVID-19 and diabetes [15,16]. Indeed, a meta-analysis that included a total of 12,794 adult patients suggested that diabetes protected against ARDS. The Lung Injury Prediction Score (LIPS) was created in 2011 with the intent to facilitate the design and conduct of ARDS prevention studies. As per this LIPS scoring system they have given DM minus one point, specifying that DM has a preventive role in developing ARDS. Although the exact mechanism is not known, one possible explanation is that diabetic patients have impaired activation of the inflammatory cascade in the lungs [17]. Other research studies have shown that patients with DM with sepsis, pneumonia, trauma, aspiration, or massive transfusion were associated with lower rates of developing ARDS [18]. With the increasing concerns about the COVID-19 pandemic and lack of data, it’s necessary to understand the impact of uncomplicated DM on ARDS among COVID-19 patients, and this study will focus on providing more knowledge and details.

## Materials and methods

A retrospective recorded based cohort study was conducted in Asher Center Hospital (ACH) (IRB: RES-18-06-2021) during the period from July 10, 2021 to Jan 15, 2022 where confirmed inpatient COVID-19 cases in Aseer region, Saudi Arabia, were classified into two groups. First group were diabetic patients without any diabetes related complications and confirmed for COVID-19 infection (diabetes group). The second group were confirmed COVID-19 patients free of any chronic health problem (healthy cases). Diabetic patients with any associated complications and non-diabetic COVID-19 patients with any chronic health problem were excluded. Also, patients with incomplete files for clinical and relevant data were excluded. Data extracted using pre-structured data extraction sheet to avoid data extraction error and confirm data consistency. Extracted data included patients’ diabetes status, medical history, socio-demographic data, COVID-19 infection data and vaccination, experienced signs and symptoms, respiratory distress, need for hospitalization, need for mechanical ventilation and ICU admission.

Data analysis

After data were extracted, it was revised, coded, and fed to statistical software SPSS version 22 (IBM Corp., Armonk, NY, USA). All statistical analysis was done using two tailed tests. P value less than 0.05 was statistically significant. Comparative analysis based on frequency and percent distribution was done for all variables including study groups socio-demographic data, COVID-19 infection data, COVID-19 vaccination, experienced signs and symptoms, and hospitalization data for the two groups. Also, the two study groups were compared regarding the needed for mechanical ventilation and ICU admission due to acute respiratory distress syndrome totally then by patients' age (as it is expected to confound the relation under study). Relations were tested using Pearson chi-square test and exact probability test for small frequency distributions.

## Results

The study included 144 patients with uncomplicated diabetes and 323 patients without comorbidities who had COVID-19 infection. The mean age of the diabetic group was 65.4 ± 12.9 years old compared to 40.2 ± 11.9 years old for the healthy group with a statistically significant difference (P=.001). A total of 73.6% of the diabetic group were males compared to 73.1% of the healthy group (P=.902). An exact 75% of the diabetic group was Saudi versus 64.7% of the healthy group (P=.028) (Table 1).

**Table 1:** Personal characteristics of covid infected study groups, Aseer region, Saudi Arabia P: Pearson X2 test $: Exact probability test * P < 0.05 (significant)

Personal data	Group				p-value
	Diabetic		Normal		
	No	%	No	%	
Age in years					.001*^$^
18-29	2	1.4%	24	7.4%	
30-49	47	32.6%	190	58.8%	
50-79	92	63.9%	104	32.2%	
80+	3	2.1%	5	1.5%	
Gender					.902
Male	106	73.6%	236	73.1%	
Female	38	26.4%	87	26.9%	
Nationality					.028*
Saudi	108	75.0%	209	64.7%	
Non-Saudi	36	25.0%	114	35.3%	

COVID-19 infection and vaccination data among study groups, Aseer region, Saudi Arabia is shown in Table 2. Only one case in the diabetic group was vaccinated against COVID-19 during the study period versus two cases in the healthy group (P=.925). Also, 14 (9.7%) in the diabetic group had exposure to confirmed COVID-19 cases in comparison to 44 (13.6%) healthy cases (P=.238). A total of four (2.8%) diabetic cases travelled abroad two weeks before study onset compared to six (1.9%) healthy cases (P=.526). Additionally, 12 (8.3%) of the diabetic cases had COVID-19-related complications versus 41 (12.7%) healthy cases (P=.170). Hospitalization due to COVID-19 infection was reported among all diabetic cases versus 319 (98.8%) healthy cases (P=.180). 

**Table 2:** Covid-19 infection and vaccination data among study groups, Aseer region, Saudi Arabia P: Pearson X2 test $: Exact probability test

Covid-19 data	Group				p-value
	Diabetic		Normal		
	No	%	No	%	
Have you been vaccinated against Covid 19?					.925^$^
Yes	1	.7%	2	.6%	
No	143	99.3%	321	99.4%	
Has your contact been confirmed with Covid 19?					.238
Yes	14	9.7%	44	13.6%	
No	130	90.3%	279	86.4%	
Have you come back from abroad in the past 14 days?					.526^$^
Yes	4	2.8%	6	1.9%	
No	140	97.2%	317	98.1%	
Did you get complications from Covid 19?					.170
Yes	12	8.3%	41	12.7%	
No	132	91.7%	282	87.3%	
Were you hospitalized because of COVID 19?					.180
Yes	144	100.0%	319	98.8%	
No	0	0.0%	4	1.2%	

Experienced symptoms data among study groups, Aseer region, Saudi Arabia, is shown in Table 3. All reported symptoms were nearly reported by the same frequency among the two study groups, especially for fever, sore throat, headache, sweating, loss of smell and taste, tachycardia, and disturbances in consciousness. Only acute onset of respiratory distress was more reported among healthy cases (10.2%) than among diabetic cases (5.6%) but all had no significant difference (P>0.05 for all).

**Table 3:** Experienced symptoms data among study groups, Aseer region, Saudi Arabia P: Pearson X2 test $: Exact probability test

Symptoms	Group				p-value
	Diabetic		Normal		
	No	%	No	%	
Fever (38 degrees Celsius)	100	69.4%	224	69.3%	.984
Sore throat	11	7.6%	24	7.4%	.937
Runny nose	7	4.9%	10	3.1%	.347
Dry coughing	111	77.1%	258	79.9%	.494
Shortness of breath	129	89.6%	296	91.6%	.473
Vomiting	15	10.4%	45	13.9%	.294
Nausea	9	6.3%	30	9.3%	.273
Diarrhea	23	16.0%	64	19.8%	.325
Headaches	27	18.8%	60	18.6%	.964
Loss of appetite	4	2.8%	16	5.0%	.284
Sweating	1	.7%	3	.9%	.800^$^
lost smell and taste	7	4.9%	16	5.0%	.966
Tiredness (general stress)	37	25.7%	92	28.5%	.534
Disturbances in consciousness	2	1.4%	5	1.5%	.896^$^
Increased heart rate	6	4.2%	14	4.3%	.934
Abdominal pain	8	5.6%	15	4.6%	.674
Chest pain	14	9.7%	30	9.3%	.882
Respiratory Distress	10	6.9%	24	7.4%	.852
Acute onset within 1 week	8	5.6%	33	10.2%	.100

Acute respiratory distress syndrome among study groups, Aseer region, Saudi Arabia, is shown in Table 4. Five (3.5%) diabetic cases needed mechanical ventilation (MV) during hospitalization compared to 23 (7.1%) healthy cases with no statistical significance (P=.125). Also, 12 (8.3%) diabetic cases were admitted to ICU versus 42 (13%) healthy cases (P=.145). 

**Table 4:** Acute respiratory distress syndrome (ARDS) among study groups, Aseer region, Saudi Arabia P: Pearson X2 test $: Exact probability test

ARDS data	Group				p-value
	Diabetic		Normal		
	No	%	No	%	
Have you been put on mechanical ventilation during your hospitalization?					.125
Yes	5	3.5%	23	7.1%	
No	139	96.5%	300	92.9%	
ICU admission					.145
Yes	12	8.3%	42	13.0%	
No	132	91.7%	281	87.0%	

Acute respiratory distress syndrome among study groups by their age, Aseer region, Saudi Arabia, is shown in Table 5. A total of 3.2% of diabetic cases aged more than 50 years needed MV compared to 10.1% of healthy cases with recorded statistical significance (P=.047). Also, 7.4% of diabetic cases needed ICU admission compared to 13.8% of healthy cases (P=.049).

**Table 5:** Acute respiratory distress syndrome (ARDS) among study groups by their age, Aseer region, Saudi Arabia P: Exact probability test * P < 0.05 (significant)

Age in years	ARDS	Group				p-value
		Diabetic		Normal		
		No	%	No	%	
< 50	Mechanical ventilation					.668
	Yes	2	4.1%	12	5.6%	
	No	47	95.9%	202	94.4%	
	ICU admission					.641
	Yes	5	10.2%	27	12.6%	
	No	44	89.8%	187	87.4%	
> 50	Mechanical ventilation					.047*
	Yes	3	3.2%	11	10.1%	
	No	92	96.8%	98	89.9%	
	ICU admission					.049*
	Yes	7	7.4%	15	13.8%	
	No	88	92.6%	94	86.2%	

## Discussion

Severe COVID-19 infection may cause viral pneumonia through infection by SARS-CoV-2, causing ARDS. ARDS clinical features can be observed as a combination of the two processes, namely viral pneumonia and ARDS [19-20]. Some research has shown that patients with DM with sepsis, pneumonia, trauma, aspiration, or massive transfusion were associated with lower rates of developing ARDS [18]. Some studies showed that diabetes may be related to lower development of ARDS among patients having at least one prompting ARDS condition such as sepsis [21-26], while one study reported diabetes associated with a higher risk of ARDS in postsurgical patients [27]. Though, a third study estimated that diabetes was not associated with development of ARDS in an unselected ICU population [28].

The current study aimed to determine the effect of uncomplicated diabetes mellitus on ARDS among COVID-19 patients in the Aseer region. The study results showed that there was no significant difference between the study groups regarding COVID-19 infection-related data which is a very important and promising result as any reported significant difference may be a confounder for the relation between uncomplicated diabetes status and developing ARDS. This means any differences between the study groups were attributed to their diabetes status only. The other finding which strengthened this conclusion was that there also were no significant differences in the experienced symptoms among the study groups. On the other hand, the current study findings revealed that there was no significant difference between healthy COVID-19-infected patients and diabetic patients regarding the need for mechanical ventilation, which was higher among healthy cases than among diabetic patients. Also, ICU admission was insignificantly higher among healthy COVID-19 patients than among diabetic COVID-19 cases which was low in both groups. The age as an expected confounder was adjusted using stratified analysis where ICU admission was significantly higher among normal COVID-19 patients than uncomplicated diabetic infected cases (13.8% vs. 7.4%) among patients aged above 50 years. These findings stand beside the theory of protective effect for uncomplicated diabetes against ARDS in general and during COVID-19 infection. 

Type 2 diabetes is more frequent in older age with higher risk for chronic kidney disease, cardiovascular disease, and other chronic co-morbidities. It has not been recognised whether diabetes itself independently affects risk of progression from mild to severe complications in COVID-19, or if associated co-morbidities including hypertension, obesity, and cardiovascular or kidney disease may be the contributing factors. These conditions are improbable to be modified after diagnosis of infection, but other issues of diabetes may be adaptable. Glucose control is important in hospitalized patients and can be modified [29].

## Conclusions

In conclusion, there is a great controversy regarding the effect of diabetes on the progression of COVID-19 infection to ARDS. The current study showed that there was no significant difference between diabetic and healthy COVID-19-infected cases regarding ARDS-related clinical factors, mainly need for ICU admission and mechanical ventilation. Studies that meticulously assess the independent associations of diabetes and glycemic control with outcomes of COVID-19 in the intensive care setting are needed.

## References

[1] Walls AC, Park YJ, Tortorici MA, Wall A, McGuire AT, Veesler D (2020). Structure, funcion, and an1genicity of the SARS-CoV-2 spike glycoprotein. Cell.

[2] Velavan TP, Meyer CG (2020). The COVID-19 epidemic. Trop Med Int Health.

[3] Gibson PG, Qin L, Puah SH (2020). COVID-19 acute respiratory distress syndrome (ARDS): clinical features and differences from typical pre-COVID-19 ARDS. Med J Aust.

[4] Mann A, Early GL (2012). Acute respiratory distress syndrome. Mo Med.

[5] Tomashefski JF (20001). Pulmonary pathology of acute respiratory distress syndrome. Clin Chest Med.

[6] Sheu CC, Gong MN, Zhai R (2010). Clinical characteristics and outcomes of sepsis-related vs non-sepsis-related ARDS. Chest.

[7] Bendib I, de Chaisemartin L, Mekontso Dessap A, Chollet-Martin S, de Prost N (2019). Understanding the role of neutrophil extracellular traps in pa1ents with severe pneumonia and ARDS. Chest.

[8] (2004). Diagnosis and classification of diabetes mellitus. Diabetes Care.

[9] (2008). The Epidemiology of Diabetes Mellitus.

[10] Muniyappa R, Gubbi S (2020). COVID-19 pandemic, coronaviruses, and diabetes mellitus. Am J Physiol Endocrinol Metab.

[11] Onder G, Rezza G, Brusaferro S (2020). Case-fatality rate and characteristics of patients dying in relation to COVID-19 in Italy. JAMA.

[12] Zhou F, Yu T, Du R (2020). Clinical course and risk factors for mortality of adult inpatients with COVID-19 in Wuhan, China: a retrospective cohort study. Lancet.

[13] Guan WJ, Ni ZY, Hu Y (2020). Clinical characteristics of coronavirus disease 2019 in China. N Engl J Med.

[14] Wu Z, McGoogan JM (2020). Characteristics of and important lessons from the coronavirus disease 2019 (COVID-19) outbreak in China: summary of a report of 72 314 cases from the Chinese Center for Disease Control and Prevention. JAMA.

[15] Zhu L, She ZG, Cheng X (2020). Association of blood glucose control and outcomes in patients with COVID-19 and pre-existing type 2 diabetes. Cell Metab.

[16] Bode B, Garrett V, Messler J, McFarland R, Crowe J, Booth R, Klonoff DC (2020). Glycemic characteristics and clinical outcomes of COVID-19 patients hospitalized in the United States. J Diabetes Sci Technol.

[17] Odeyemi Y, De Moraes AG, Gajic O (2020). What factors predispose patients to acute respiratory distress syndrome?. Evid Based Pract Crit Care.

[18] Yu S, Christani DC, Thompson BT, Bajwa EK, Gong MN (2013). Role of diabetes in the development of acute respiratory distress syndrome. Crit Care Med.

[19] Tzotzos SJ, Fischer B, Fischer H, Zeitlinger M (2020). Incidence of ARDS and outcomes in hospitalized patients with COVID-19: a global literature survey. Crit Care.

[20] Yu T, Cai S, Zheng Z (2020). Association between clinical manifestations and prognosis in patients with COVID-19. Clin Ther.

[21] Gong MN, Thompson BT, Williams P, Pothier L, Boyce PD, Christiani DC (2005). Clinical predictors of and mortality in acute respiratory distress syndrome: potential role of red cell transfusion. Crit Care Med.

[22] Iscimen R, Cartin-Ceba R, Yilmaz M, Khan H, Hubmayr RD, Afessa B, Gajic O (2008). Risk factors for the development of acute lung injury in patients with septic shock: an observational cohort study. Crit Care Med.

[23] Gong MN, Bajwa EK, Thompson BT, Christiani DC (2010). Body mass index is associated with the development of acute respiratory distress syndrome. Thorax.

[24] Esper AM, Moss M, Martin GS (2009). The effect of diabetes mellitus on organ dysfunction with sepsis: an epidemiological study. Crit Care.

[25] Trillo-Alvarez C, Cartin-Ceba R, Kor DJ (2011). Acute lung injury prediction score: derivation and validation in a population-based sample. Eur Respir J.

[26] Gajic O, Dabbagh O, Park PK (2011). Early identification of patients at risk of acute lung injury: evaluation of lung injury prediction score in a multicenter cohort study. Am J Respir Crit Care Med.

[27] Kor DJ, Warner DO, Alsara A (2011). Derivation and diagnostic accuracy of the surgical lung injury prediction model. Anesthesiology.

[28] Koh GC, Vlaar AP, Hofstra JJ (2012). In the critically ill patient, diabetes predicts mortality independent of statin therapy but is not associated with acute lung injury: a cohort study. Crit Care Med.

[29] Selvin E, Juraschek SP (2020). Diabetes epidemiology in the COVID-19 pandemic. Diabetes Care.

